# Resistance strategies of *Phragmites australis* (common reed) to Pb pollution in flood and drought conditions

**DOI:** 10.7717/peerj.4188

**Published:** 2018-01-03

**Authors:** Na Zhang, Jinwei Zhang, Zhiqiang Li, Jing Chen, Zhenhua Zhang, Chunsheng Mu

**Affiliations:** 1 Institute of Agricultural Resource and Environment, Jiangsu Academy of Agricultural Sciences, Nanjing, China; 2 Key Laboratory for Palygorskite Science and Applied Technology of Jiangsu Province, Huaiyin Institute of Technology, Huaian, China; 3 Key Laboratory of Vegetation Ecology, Ministry of Education, Institute of Grassland Science, Northeast Normal University, Changchun, China; 4 Academy of Climate Change and Public Policy, Nanjing University of Information Science and Technology, Nanjing, China

**Keywords:** *Phragmites australis*, Flooded treatment, Photosynthesis, Drought, Clonal growth, Biomass allocation

## Abstract

Resistance strategies of clonal organs, and parent and offspring shoots of *Phragmites australis* (common reed) to heavy metal pollution in soils are not well known. To clarify the tolerance or resistance strategies in reeds, we conducted a pot experiment with five levels of Pb concentration (0∼4,500 mg kg^−1^) in flood and drought conditions. Lead toxicity had no inhibitory effect on the number of offspring shoots in flood environment; however, biomass accumulation, and photosynthetic and clonal growth parameters were inhibited in both water environment. At each treatment of Pb concentration, offspring shoots had greater biomass and higher photosynthesis indicators than parent shoots. The lowest Pb allocation was found in rhizomes. More of the Pb transported to above-ground parts tended to accumulate in parent shoots rather than in offspring shoots. Biomass and photosynthesis of offspring shoots, rhizome length, and the number of buds, rhizomes and offspring shoots in the flooded treatment were significantly greater than those in the drought treatment. Our results indicated that the tolerance strategies used by reeds, including higher biomass accumulation and photosynthesis in offspring shoots, low allocation of Pb in rhizomes and offspring shoots, and stable clonal growth, maintained the stability of population propagation and productivity, improving the resistance of reeds to Pb pollution in flood environment. However, the resistance or tolerance was significantly reduced by the synergistic effect of Pb and drought, which significantly inhibited biomass accumulation, photosynthesis, and clonal growth of reeds.

## Introduction

Heavy metal contamination in soils has been a serious problem, including decreased crop yields, biomass accumulation, and inhibited plant physiological metabolism in many areas around the world (Mohammed, Kapri & Goeland, 2011; Jiang et al., 2014). Lead (Pb), a heavy metal that is moderately immobile in soils, is a major source of soil contamination. Soils containing ranges of Pb concentration of 10∼30 and 30∼100 mg kg^−1^ are considered uncontaminated and slightly contaminated, respectively (Davies, 1990). When Pb concentrations reach 100∼500 mg kg^−1^ soil, plants exhibit symptoms of toxicity (Kabata-Pendias & Pendias, 1984) including inhibition of antioxidant enzyme activities, increase in lipid peroxidation, disturbance in mineral nutrient balance, and inhibition of photosynthesis (Gopal & Rizvi, 2008; Islam et al., 2008; Hu et al., 2012; Wang et al., 2012), which affect plant growth and survival.

Biomass allocation is a vital plant mechanism for coping with adverse environment (Liu et al., 2014). For some herbaceous perennial plants, they could produce offspring shoots that develop from below-ground buds attached to rhizomes or other perennial organs (Rogers & Hartnett, 2001; Zhang et al., 2009). The above-ground population of these plant species is comprised of both parent and offspring shoots, which determine the above-ground biomass or productivity (Harper, 1977; Benson & Hartnett, 2006). A considerable body of literature has focused on the effects of external factors on above- and below-ground biomass allocation (Pezeshki, DeLaune & Meeder, 1997; Asch et al., 2005; Ryser & Emerson, 2007). However, biomass allocation to clonal modules (including rhizomes, parent and offspring shoots) has not been addressed. Specifically, little information is available regarding the allocation of above-ground productivity to parent and offspring shoots in perennial plants. It is rare to systematically research on the biomass allocation in clonal module and other organs of perennial plants under disturbances.

The plant growth and material accumulation are directly correlated with photosynthesis, the energy source of plant development. Photosynthesis is also considered to be one of the most sensitive metabolic processes to Pb toxicity (Sharma & Dubey, 2005). The reasons may include closure of stomata, damage to chloroplast ultrastructure, inhibition of chlorophyll synthesis due to increasing chlorophyllase activity, obstruction of electron transport, and restriction of the enzyme activities in the Calvin cycle (Sharma & Dubey, 2005; Islam et al., 2008; Hu et al., 2012). Such changes in key photosynthetic processes eventually lead to inhibition of plant growth and biomass accumulation. At present, more attentions focus on the photosynthetic response of whole population (Pagter, Bragato & Brix, 2005; Liu et al., 2009), while few researchers concern about how the photosynthesis of parent and offspring shoots responds to heavy metal pollution, and how the responses affect the population productivity of perennial plants.

Population establishment from seeds occurs infrequently in perennial plants. The above-ground shoots mainly originate from below-ground buds on rhizomes or other perennial organs (Benson, 2001; Nishihiro et al., 2004). The below-ground bud bank and its resprouting capability are closely related to the growth of rhizomes, which provide resources for growth of bud and offspring shoots. Therefore, clonal growth of rhizomes and buds partly exhibit the resistance ability of perennial plants to disturbance. The importance of bud bank under undisturbed conditions has been established. For example, below-ground bud bank dynamics and vegetative reproduction regulate population density and productivity of perennial plants (Benson, Hartnett & Mann, 2004; Benson & Hartnett, 2006; Dalgleish & Hartnett, 2006; Wang et al., 2010). Large reserve population of below-ground buds may improve the resistance to and the recovery rate of above-ground plants following fire, drought, heavy grazing, or other environmental stresses (Hartnett, Setshogo & Dalgleish, 2006; Fernandes et al., 2008; Mony, Puijalon & Bornette, 2011). However, the survival and vegetative propagation of rhizomes may be hindered when plants are exposed to long-term heavy metal contamination (Fürtig et al., 1999), contributing to species decline. To date, little research has focused on the effect of heavy metals on the below-ground reproductive structure (such as rhizomes and buds), especially for aquatic plants (Zhang et al., 2015). More information is needed about the growth of below-ground clonal organs and the resprouting capacity of buds in heavy metal-contaminated environment.

*Phragmites australis* is a typical rhizomatous perennial plant with high tolerance to a variety of environmental conditions. The species has been extensively used in the remediation of wetlands containing heavy metals and other pollutants. Wetlands are usually subjected to seasonal fluctuations in water levels, including flooding and drought. We conducted a pot experiment with *Phragmites australis* along a gradient of five Pb concentrations (0, 500, 1,500, 3,000, and 4,500 mg kg^−1^) in simulated flood and drought environments. The objectives of our study were to determine: (i) how Pb affects biomass allocation into organs, and the response drivers under flood and drought conditions, (ii) how Pb and presence of water affect photosynthesis of parent and offspring shoots, (iii) how the clonal growth (buds, rhizomes and offspring shoots) responds to Pb in flood and drought environment, and to investigate (iv) the resistance strategies to Pb pollution in terms of biomass allocation, photosynthesis, and clonal growth of *P. australis* under flood and drought conditions.

## Materials and Methods

### Preparation of experiment material

THE 1,000 mature panicles of wild *P. australis* were randomly collected in paddy wetland located west of Changchun City, Jilin Province, China (125°1′E, 43°56′N, 188 m a.s.l.) in October 2013; the paddy wetland is open after harvesting. Seeds were obtained by removing lemmas from air-dried panicles. Seeds were then sown in plastic tubs (80 × 50 × 30 cm) containing 20 cm of humus soil. These plastic tubs were then put in transparent greenhouse with natural alternating temperature 15∼25 °C.

Soils for Pb-spiking were collected from the surface layer (0–20 cm) of the wetland in the dry period. The collected fresh soil was mixed homogeneously and allowed to air dry. Soil analysis yielded total nitrogen at 6.9%, organic carbon at 0.4%, pH at 8.6, electronic conductivity at 91 µs cm^−1^, and field capacity at 200 g kg^−1^. Analytical methods were taken from Lu (2000). After air-drying and passing through a 1 mm sieve, specified concentrations of Pb(NO_3_)_2_ solution were added into soil and thoroughly mixed, obtaining five levels of Pb (0, 500, 1,500, 3,000, and 4,500 mg kg^−1^). Spiked soils were transferred into 60 plastic pots (30 cm diameter × 35 cm height; 10 kg soil pot^−1^), and kept in dark in a ventilated room for 45 days. Two grams of soil from each pot was then sampled and analyzed for Pb concentration. The actual concentrations of Pb in the spiked soils were measured to be 12.42 ± 0.43, 525 ± 5.87, 1,554 ± 39.66, 3,137 ± 128.74, 4,568 ± 159.89 mg Pb kg^−1^.

### Experimental design

Fifty days after seed-sowing, 20 uniformly-sized plants, ∼8 cm high and with four or five leaves, were transplanted into each of the pots with spiked soil. After five days, we removed weak plants to keep 10 healthy seedlings in each pot.

The experiment was arranged as a random design, including two levels of water and five levels of Pb treatments, for a total of ten treatments and six replicate pots per treatment. Pots exposed to flooded treatment were directly watered with deionized water once a day, keeping a 3–5 cm water layer above the contaminated soil surface. The drought treatments were kept in soil water levels at 50–55% of field capacity using an electronic scale checked at 5:00 pm each day. The experiment was conducted in a greenhouse at Northeast Normal University, Jilin City, China (125°19′E, 43°51′N, 236 m a.s.l.), and lasted 90 days.

### Determination of clonal growth and biomass

After 90 days, randomly selected four pots from six replicate pots. plants in each selected pot were destructively sampled, and each plant was separated from the contaminated soil along with its root system. We then immediately counted the total number of buds >1 mm, rhizomes and offspring shoots, and measured the total length of rhizomes and roots on parent shoots; these parameters were expressed per parent shoot. Finally, each plant was washed gently with tap and three times with deionized water, and divided into root, rhizome, and parent and offspring shoots. These tissues were oven-dried (75 °C) to constant weights, and dry biomass was recorded. The dried tissues were used to analyze for Pb.

### Measurement of photosynthetic parameters

Different parameters of photosynthesis, such as net photosynthetic rate (P_n_), stomatal conductance (g_s_), intercellular CO_2_ concentration (C_i_), and transpiration rate (E) were determined with an LI-6400 gas exchange system (LI- 6400XT; Li-Cor Inc., Lincoln, NE, USA) after 60 days of treatment. Twenty-four of the youngest available fully expanded leaves of parent or offspring shoots (six leaves per pot, selected four pots per treatment) were selected randomly for determining photosynthesis. Average values of four leaves per pot were considered as one replicate. The photosynthetic parameters of parent shoots in drought treatment were not determined due to their stunted growth or death.

### Pb concentration determination

Soil samples were digested in a solution of 5:1:1 HNO_3_:HCLO_4_:HF (v/v) in a microwave oven (ANALYX, CEM Mars5; CEM Corporation, Matthews, NC, USA). Plant tissues with added 5:1 HNO_3_: HCLO_4_ (v/v) were digested by far-infrared thermostatic electric furnace (BXS12-LWY-84B; Jiangsu Shenglan Equipment Manufacturing Co., Ltd., Jiangsu, China). The soil and plant standard sample (CRM Soil, GBW07403 and Spinach, GSB-6) made by the Chinese Academy of Geological Science Institute of Geochemical Exploration was also digested and its Pb contents determined. The concentrations of Pb were determined using a Graphite Furnace Atomic Absorption Spectroscopy (Varian SpectrAA Z220; Varian, Palo Alto, CA, USA) after appropriate dilution.

### Statistical analysis

We used a two-way ANOVA to evaluate the effects of Pb level, water level, and the interaction of Pb and water levels on biomass, photosynthesis, clonal growth parameters, and Pb concentrations in organs. A separate ANOVA was then used to evaluate the effects of Pb under the same water level. Meanwhile, an independent sample *T*-test was used to evaluate the effect of water treatments on these parameters under the same Pb level. All statistical analyses were performed with the SPSS v.13.0 statistical package (SPSS Inc., Chicago, IL, USA), and multiple comparisons of means were performed using LSD tests at an alpha level of 0.05 when ANOVA results were significant. The figures were plotted with SIGMAPLOT 11.0 (Systat Software, Inc., San Jose, CA, USA).

## Results

### Biomass allocation

**Figure 1:**
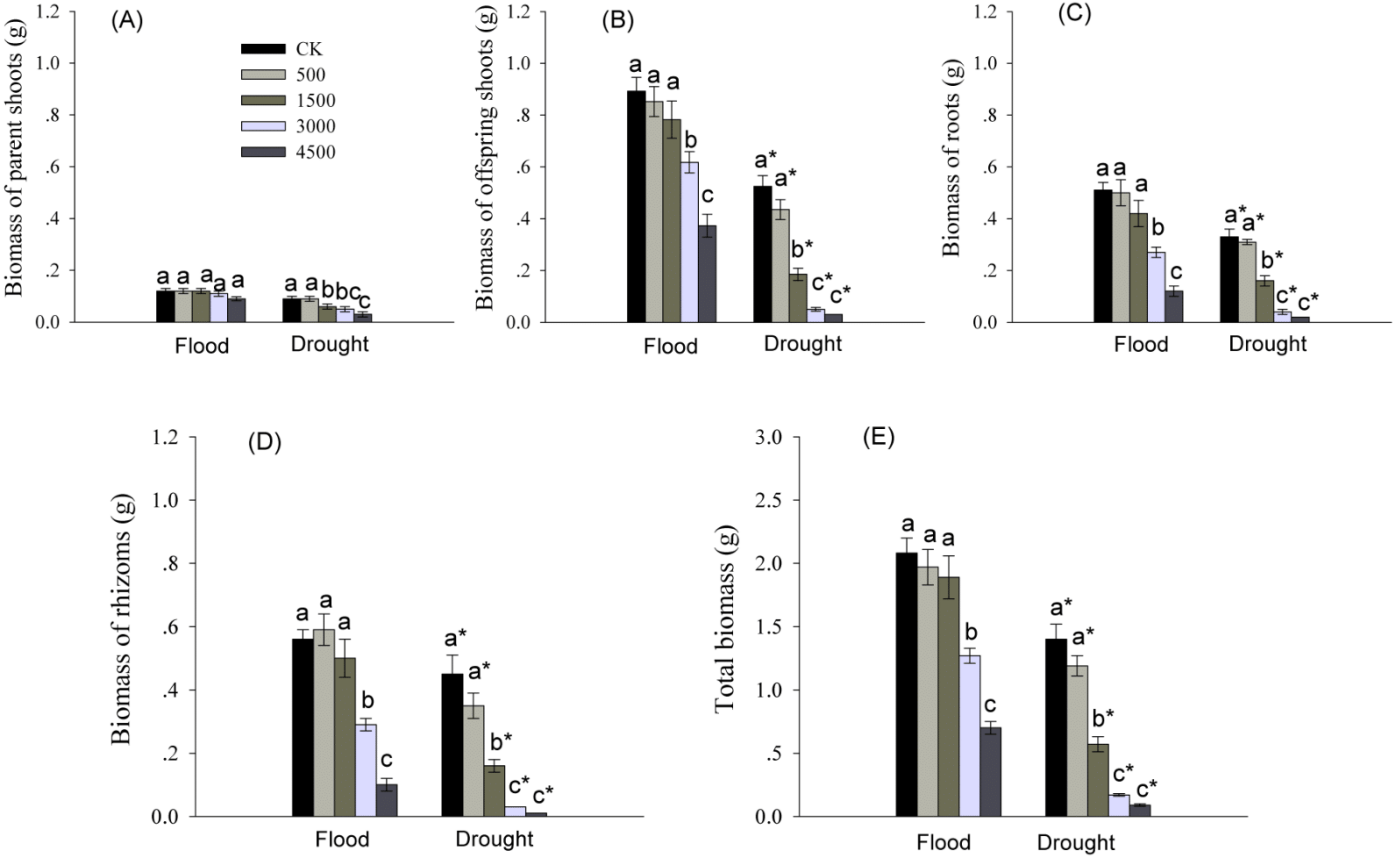
Biomass in different organs of *Phragmites australis* subjected to Pb contamination in flood and drought conditions. (A) indicates biomass of parent shoots; (B) indicates biomass of offspring shoots; (C) indicates root biomass; (D) indicates rhizome biomass; (E) indicates total biomass. Different lowercase letters indicate significant differences (*P* ≤ 0.05) among Pb levels (within a water level), an asterisk indicates a significant difference (*P* ≤ 0.05) between flood and drought treatment (within a Pb level).

Biomass of various organs was significantly affected by Pb and water treatments (Table 1). Biomass of roots, rhizomes, and offspring shoots, and total biomass were significantly reduced by higher levels of Pb in both water treatments (Fig. 1). Biomass of offspring shoots was 3∼7 times greater than that of parent shoots at the same Pb level in flood conditions (Figs. 1A, 1B). However, biomass of different organs and total biomass of reeds grown in dry conditions were less than these in flood condition at each Pb level (Fig. 1).

**Table 1:** Results of Two-way ANOVA of the effect of Pb and water stress on biomass, photosynthesis, clonal growth, and Pb concentrations in organs of *Phragmites australis*.

	Two-way ANOVA					
	Pb		Water		Pb × Water	
	F	P	F	P	F	P
**Biomass (g)**						
Root (g)	63.55	<0.001	124.45	<0.001	2.56	0.06
Rhizome (g)	55.76	<0.001	77.79	<0.001	4.41	<0.01
Parent shoot (g)	7.31	<0.001	39.32	<0.001	7.48	<0.001
Daughter shoot (g)	42.52	<0.001	270.3	<0.001	6.95	<0.001
**Clonal growth parameters**						
Root length (cm)	184.571	<0.001	273.946	<0.001	4.605	<0.01
Rhizome length (cm)	65.735	<0.001	126.045	<0.002	5.271	<0.01
No. of rhizomes (no. plant^−1^)	92.408	<0.001	207.906	<0.003	8.108	<0.001
No. of buds (no. plant^−1^)	42.807	<0.001	51.986	<0.004	6.879	<0.001
No. of daughter shoots (no. plant^−1^)	12.658	<0.001	442.488	<0.005	4.293	<0.01
**Photosynthetic parameters**						
A (µmolCO_2_ m^−2^*s*^−1^)	12.69	<0.001	20.38	<0.001	6.92	<0.001
G_s_ (mmolH_2_O m^−2^s^−1^)	16.9	<0.001	95.36	<0.001	3.8	<0.01
C_i_ (µmolCO_2_mol^−1^)	9.8	<0.001	111.35	<0.001	1.54	0.23
E (mmolH_2_O m^−2^s^−1^)	6.08	<0.001	88.8	<0.001	1.07	0.38
**Pb concentration in plants**						
Root systems (mg kg^−1^)	472.06	<0.001	1,061.42	<0.001	166.3	<0.001
Parent shoot (mg kg^−1^)	3,304.2	<0.001	369.17	<0.001	434.26	<0.001
Daughter shoot (mg kg^−1^)	2,392.36	<0.001	42.83	<0.001	31.27	<0.001

Moreover, we observed that the parent shoots treated with 4,500 mgPb kg^−1^ under flood conditions, and by Pb at >500 mgkg ^−1^ in dry conditions showed symptoms of toxicity, such as etiolated leaf and stunted growth.

### Photosynthetic response

**Figure 2:**
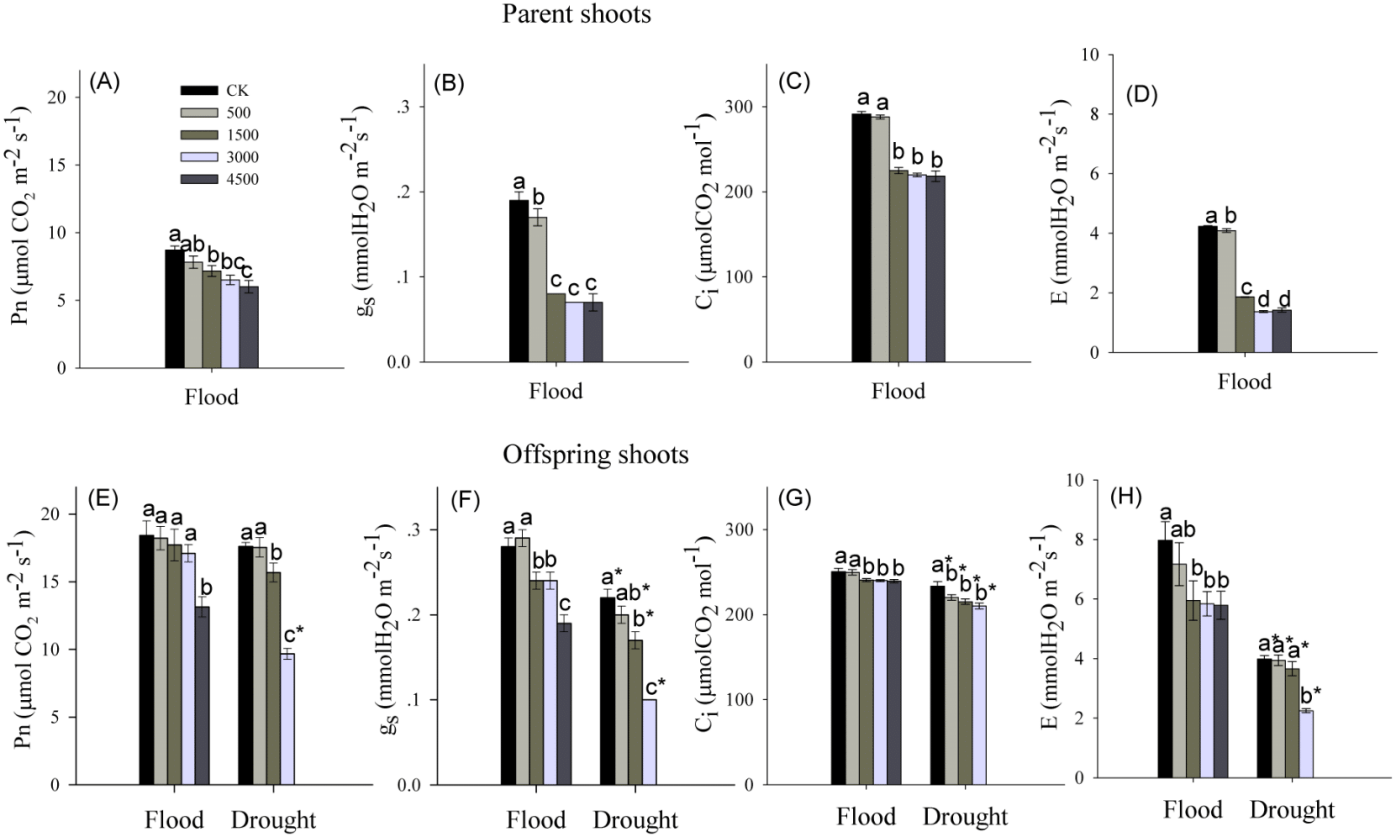
Photosynthetic parameters in parent and offspring shoots of *Phragmites australis* subjected to Pb contamination in flood and drought conditions. (A) indicates P_n_ of parent shoots, and P_n_ means net photosynthetic rate; (B) indicates g_s_ of parent shoots, and g_s_ means stomatal conductance; (C) indicates C_i_ of parent shoots, and C_i_ means intercellular CO_2_ concentration; (D) indicates E of parent shoots, and E means transpiration rate. (E) indicates P_n_ of offspring shoots; (F) indicates g_s_ of offspring shoots; (G) indicates C_i_ of offspring shoots; (H) indicates E of offspring shoots. Different lowercase letters indicate significant differences (*P* ≤ 0.05) among Pb levels (within a water level), an asterisk indicates a significant difference (*P* ≤ 0.05) between flood and drought treatment (within one Pb level).

Pb and water treatments had visible effects on the net photosynthetic rate (P_n_), intercellular CO_2_ concentration (C_i_), stomatal conductance (g_s_), and transpiration rate (E), while the interaction of Pb × Water had no effects on C_i_ and E (Table 1). The P_n_, C_i_, g_s_ and E showed a significant decrease for parent shoots treated with Pb under flooded conditions (Figs. 2A–2D). In both water levels, Pb exposure noticeably inhibited P_n_, g_s_, C_i_ and E of the offspring shoots (Figs. 2E–2H). At the same level of Pb, P_n_, C_i_, g_s_, and E of offspring shoots were greater than these of parent shoots.

### Below-ground organs and clonal growth

**Figure 3:**
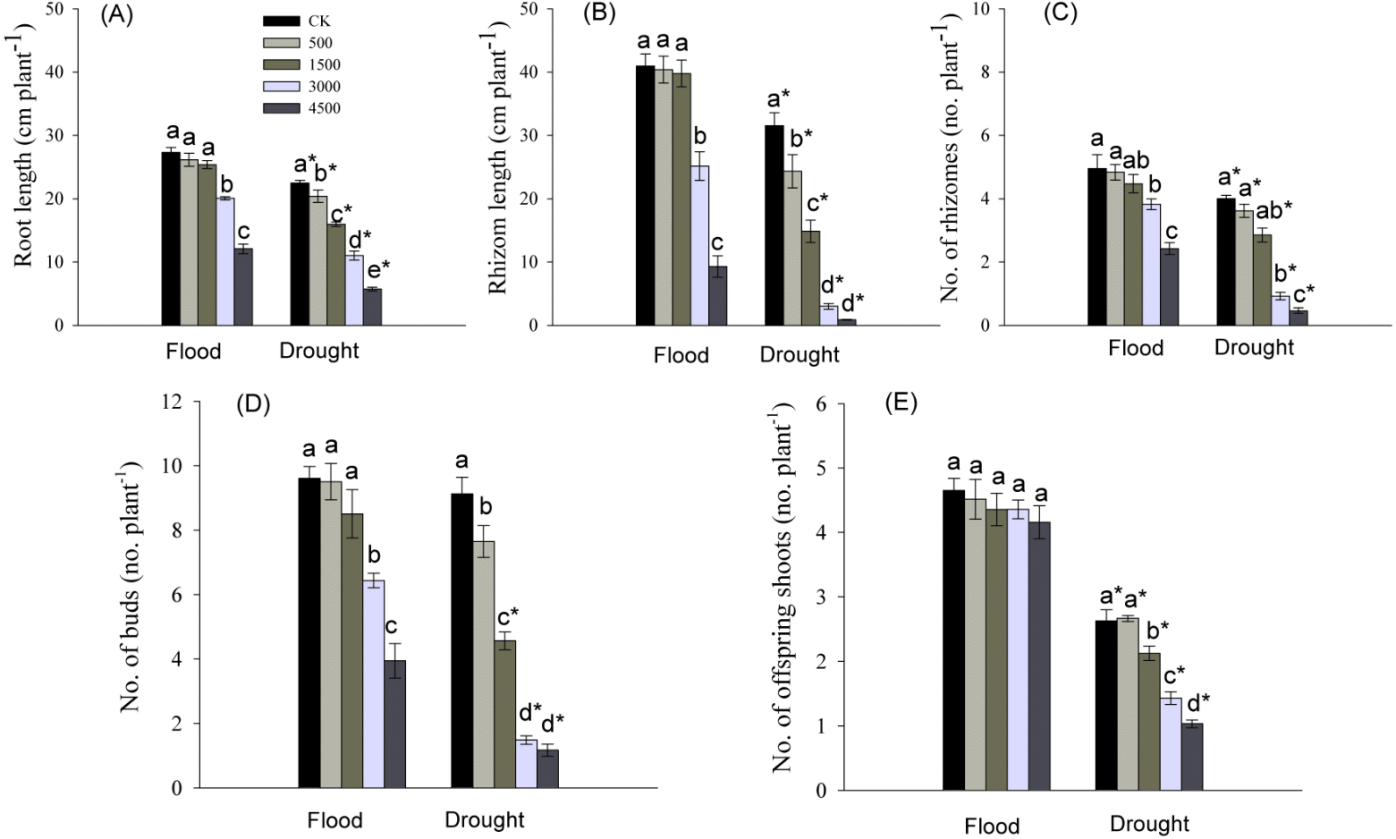
Parameters of root and clonal growth of *Phragmites australis* subjected to Pb contamination in flood and drought conditions. (A) indicates root length; (B) indicates rhizome length; (C) indicates no. of rhizomes; (D) indicates no. of buds; (E) indicates no. of offspring shoots. Different lowercase letters indicate significant differences (*P* ≤ 0.05) among Pb levels (within a water level), an asterisk indicates a significant difference (*P* ≤ 0.05) between flood and drought treatment (within one Pb level).

Root length, and number and total length of rhizomes were significantly influenced by Pb, water stress, and their interaction (Table 1). Root elongation was significantly inhibited by the higher concentrations of Pb (3,000 and 4,500 mg kg^−1^) in a flood environment, while root growth was significantly inhibited by each level of Pb in a dry environment (Fig. 3A). The significant reductions in the number and total length of rhizomes were observed at higher levels of Pb in the flooded treatment and at each Pb level in the drought treatment (Figs. 3B, 3C). The abundance and length of rhizomes in the dry environment were lower than those in a flood environment at the same Pb level (Figs. 3B, 3C).

The abundance of buds and offspring shoots was significantly affected by Pb, water stress, and Pb × Water interaction (Table 1). At both water levels, the number of buds tended to decrease with increasing Pb concentration in soil, and the decline was greater in drought than in the flooded treatment at each Pb level (Figs. 3D, 3E). The number of offspring shoots was significantly lower due to Pb additions under drought, while it was not affected by Pb in the flood environment (Fig. 3E).

### Pb concentrations in plants

**Figure 4:**
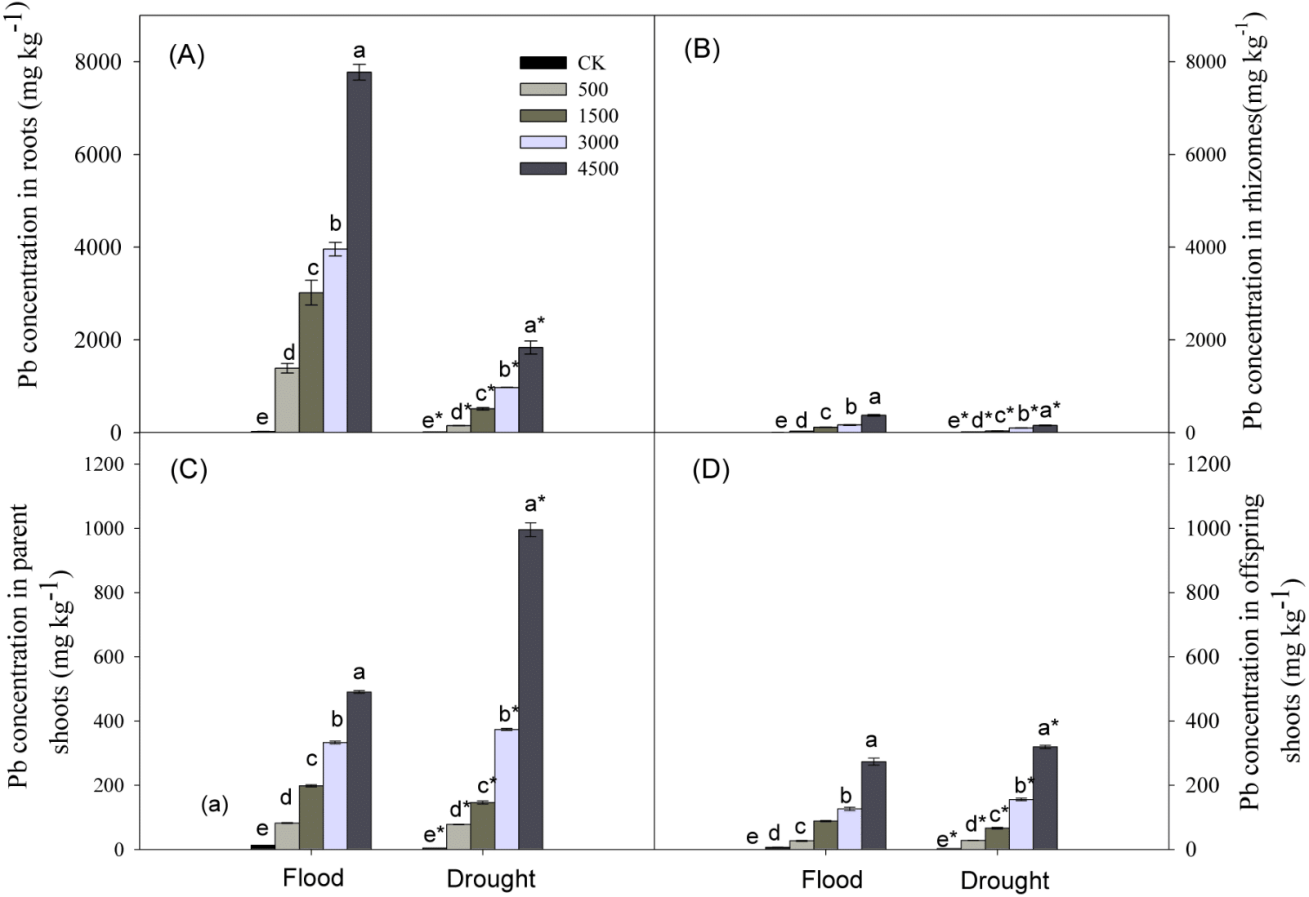
Lead concentrations in different organs of *Phragmites australis* subjected to Pb contamination in flood and drought conditions. (A) indicates Pb concentration in roots; (B) indicates Pb concentration in rhizomes; (C) indicates Pb concentration in parent shoots; (D) indicates Pb concentration in offspring shoots. Different lowercase letters indicate significant differences (*P* ≤ 0.05) among Pb levels (within a water level), an asterisk indicates a significant difference (*P* ≤ 0.05) between flood and drought treatment (within one Pb level).

Lead, water stress, and Pb × Water interaction had significant effects on Pb concentrations in plant organs (Table 1). Pb concentrations in organs increased with increasing concentrations of soil Pb. Lead concentrations in different organs followed the trend: roots >parent shoots >offspring shoots >rhizomes, and concentration of Pb in parent shoots was ∼3 times greater than that in offspring shoots (Fig. 4) at both levels of water. Lead concentrations in below-ground organs were greater under the flood compared to the drought treatment, while the reverse was true for parent or offspring shoots at Pb levels of 3,000 and 4,500 mg kg^−1^ (Fig. 4).

## Discussion

A large body of literature has shown that *P. australis* was a “root accumulator” of heavy metals (Ye et al., 1997; Ye et al., 1998; Weis & Weis, 2004; Windham, Weis & Weis, 2001; Liu et al., 2009; Hechmi et al., 2014). This explained why the highest concentration of Pb in this study was found in below-ground root systems (Fig. 4). The filtering effect of roots may be an effective strategy for protecting rhizomes and above-ground photosynthetic tissues against toxicity of heavy metals (Keller, Lajtha & Cristofor, 1998; Fürtig et al., 1999). Conversely, the highest accumulation of Pb in roots may inhibit their own growth (Fig. 1C).

Below-ground buds that are attached to rhizomes or other perennial organs are the main propagative source of above-ground shoots in perennial plants (Benson, 2001; Nishihiro et al., 2004). The lateral spread of rhizomes may expand the space from which to provide resources for regrowth of offspring shoots arising from below-ground buds after disturbance (Prach & Pyšek, 1994; Henry & Amoros, 1996; Brewer & Bertness, 1996). We found that Pb toxicity had no inhibitory effects on the number of offspring shoots, although Pb at concentrations ≥3,000 mg kg^−1^ significantly inhibited bud formation and rhizome growth in flood conditions (Figs. 3B–3D). Interestingly, we also observed that the lowest amounts of Pb were allocated to rhizomes of *P. australis* (Fig. 4), similar to the findings of our previous research (Zhang et al., 2015). Clearly, the lowest levels of Pb in rhizomes, especially in flood environments, could effectively protect clonal propagative organs, which provided energy for the growth of below-ground buds into offspring shoots, maintaining above-ground population density. Heavy metal toxicity to plants may be partly reduced by adequate water management (Rizwan et al., 2016). Conversely, the synergistic effect of Pb and drought significantly decreased rhizome growth, and the number of buds and offspring shoots in dry conditions (Figs. 3B–3E). As a result, the decline in rhizome length and the number of buds and offspring shoots was greater in the dry than in flood conditions. The significant decline in rhizome biomass with an increasing concentration of soil Pb was closely correlated with decreases in the number and length of rhizomes (Figs. 1D, 3B, 3C). Thus, low amounts of Pb in rhizomes may stabilize clonal growth, maintaining above-ground population in flood conditions. Conversely, the synergistic inhibitory effect of Pb and drought on the growth of rhizomes and buds, and on the number of offspring shoots would inhibit clonal propagation of *P. australis* under drought conditions.

Perennial clonal plants produce new shoots, defined as offspring shoots, depending mainly on the vegetative propagation by bud bank on rhizomes or other perennial organs (Klimeš et al., 1997; Rogers & Hartnett, 2001; Kliměsová & Kliměs, 2007; Klimešová & Klimeš, 2008). Therefore, above-ground biomass production of clonal plant species is determined by the contribution of parent and offspring shoots (Harper, 1977; Benson & Hartnett, 2006). Plant growth and biomass accumulation are directly correlated with photosynthesis, the source of energy for plant development. In our study, parent shoots treated with 4,500 mg Pb kg^−1^ under flooded conditions, and with >500 mg Pb kg^−1^ in drought conditions exhibited symptoms of toxicity, such as etiolated leaves and stunted growth. These phenomena may be caused by a change in photosynthetic processing, separate from stomatal closure. Specifically, the substitution of essential elements like Mg, Fe, and Mn for Pb in the chloroplast can inhibit chlorophyll synthesis, and damage the formation of grana, electron transport, and activity of PSII and Calvin cycle enzymes (Sharma & Dubey, 2005; Islam et al., 2008). Biomass of parent shoots in the flood treatment was greater than that in the drought environment (Fig. 1A). This might be caused by compensatory growth of parent shoots in flooded treatment (Zhang et al., 2015). To resist an external disturbance, resources may be shifted from parent to rhizome, bud, and offspring shoot growth at an early growth period; this would be considered an example of apical resource transport (Zhu, 2004). Over an extended period of growth, stored photosynthetic resources of offspring shoots may be transported back to parent shoots; this would be an example of basal resource transport (Zhu, 2004), which may provide resources for compensatory growth of parent shoots.

At both water levels, low biomass of offspring shoots may be due to the reduction in photosynthetic capacity at higher levels of Pb (Pb ≥ 3,000 mg kg^−1^; Figs. 1B, 2E–2H). Stomatal closure was the probable reason for the reduced photosynthesis in offspring shoots, because reduced net photosynthetic rates (P_n_) were accompanied by decreased stomatal conductance (g_s_) and transpiration rates (E), especially at high levels of Pb (Figs. 2E–2H). Several authors proposed that reduced plant photosynthesis was caused by stomatal closure rather than a direct effect of Pb on the process of photosynthesis (Bazzaz, Carlson & Rolfe, 1975; Sharma & Dubey, 2005). Although the biomass and photosynthetic rates in offspring shoots were inhibited by Pb in this study, both were greater than those in parent shoots (Figs. 1A, 1B, 2). Our results indicated that above-ground productivity was mainly due to offspring shoots. Meanwhile, biomass of offspring shoots and their photosynthetic rates were greater in flood than in dry conditions (Figs. 1A, 1B, 2). Net photosynthetic rates of offspring shoots were not unaffected by Pb levels <4,500 mg kg^−1^ in the flood environment (Fig. 2E). Therefore, offspring shoots exhibited a stronger photosynthetic tolerance to soil Pb in flood than in drought conditions, maintaining above-ground productivity. However, above-ground productivity and photosynthetic capacity of offspring shoots were significantly inhibited due to the synergetic effect of Pb and drought (Figs. 1A, 1B, 2).

Allocation of Pb to old or dead organs may serve as a defense mechanism, which protects the growth and photosynthesis in young tissues against Pb phytotoxicity (Godzik, 1993; Pietrini et al., 2010). We also found that more of the Pb transported into above-ground parts tended to accumulate in parent plants rather than in offspring shoots at both water levels (Figs. 4C, 4D). In this manner higher photosynthesis, effectively protected by low allocation of Pb into offspring shoots, may lead to greater biomass accumulation in offspring versus parent shoots, especially under flooded conditions (Figs. 1A, 1B, 2, 4C, 4D). This distribution pattern of Pb in above-ground shoots can be considered as a strategy of protecting offspring shoots from Pb toxicity by parent shoots. Moreover, the barrier function of plasma membrane probably became restrained or damaged, allowing a large amount of Pb accumulation in parent shoots subjected to 4,500 mg Pb kg^−1^ in the drought treatment (Fig. 4C).

In summary, population propagation and productivity of *P. australis* in Pb-containing soils may be maintained with the strategies of stabilized clonal growth, above-ground biomass allocation, higher photosynthesis of offspring than parent shoots, and allocation of Pb in organs, especially in flood conditions. Strategies for Pb allocation in organs included the filtering effect of roots, lowest allocation of Pb into rhizomes, and parent shoots protecting offspring shoots.

## Conclusions

We observed that the greatest proportion of population productivity was due to offspring shoots as a result of higher photosynthesis rather than to parent shoots. Low Pb allocation to offspring shoots benefited photosynthesis. Rhizomes exhibited the lowest Pb allocation of all tested organs; this may effectively protect bud resprouting, especially in flood environments. The stability of above-ground population and productivity were maintained with strategies of above-ground biomass allocation, higher photosynthesis of offspring than of parent shoots, stabilized below-ground clonal growth, and allocation of Pb to organs in flood environment. These strategies improved the resistance or tolerance ability of *P. australis* to Pb in flood conditions. Conversely, resistance or tolerance of *P. australis* was significantly reduced by the synergistic effects of Pb and drought, because biomass accumulation, photosynthesis, and clonal growth were significantly inhibited by Pb pollution in drought conditions.

##  Supplemental Information

10.7717/peerj.4188/supp-1 Data S1 Raw data

## Additional Information and Declarations

### Data Availability

The following information was supplied regarding data availability: The raw data is provided in Data S1.
